# Supplementary material for: Distinct cell-type contributions and network topography of theta-nested gamma oscillations in the medial entorhinal cortex
Source: bioRxiv. 2026 Apr 22:2026.04.21.719932. Preprint. [Version 1] doi: 10.64898/2026.04.21.719932 (PMC13131635; doi:10.64898/2026.04.21.719932)
Supplement: Supplement 1 [file NIHPP2026.04.21.719932v1-supplement-1.pdf]

bioRxiv preprint doi: https://doi.org/10.64898/2026.04.21.719932; this version posted April 22, 2026. The copyright holder for this preprint (which was not certified by peer review) is the author/funder, who has granted bioRxiv a license to display the preprint in perpetuity. It is made available under aCC-BY 4.0 International license.

Supporting Information

bioRxiv preprint doi: https://doi.org/10.64898/2026.04.21.719932; this version posted April 22, 2026. The copyright holder for this preprint (which was not certified by peer review) is the author/funder, who has granted bioRxiv a license to display the preprint in perpetuity. It is made available under aCC-BY 4.0 International license.

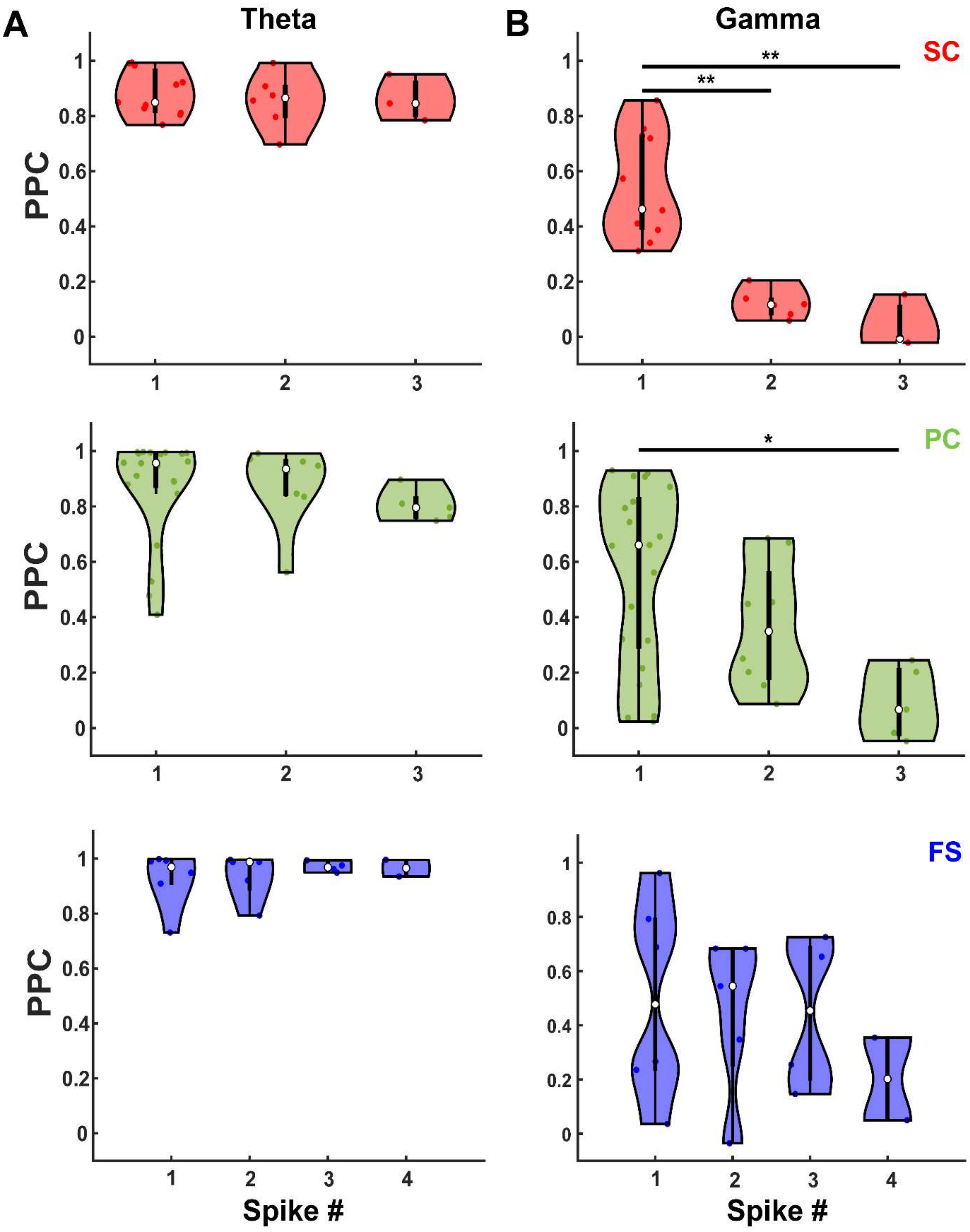

1093

1094 **Figure S1: mEC neurons are strongly phase locked to theta and moderately phase locked**
1095 **to gamma oscillations.** A) Pairwise theta phase consistency of stellate (red), pyramidal
1096 (green), and fast-spiking interneurons (blue) for each consecutive spike per theta stimulation
1097 period. All cell types and spike numbers are strongly phase locked to theta drive. B) Pairwise

bioRxiv preprint doi: https://doi.org/10.64898/2026.04.21.719932; this version posted April 22, 2026. The copyright holder for this preprint (which was not certified by peer review) is the author/funder, who has granted bioRxiv a license to display the preprint in perpetuity. It is made available under aCC-BY 4.0 International license.

gamma phase consistency of stellate (red), pyramidal (green), and fast-spiking interneurons (blue) for each consecutive spike per theta stimulation period. The first spike per theta stimulation period is moderately phase locked to LFP gamma for all cell types. Fast-spiking interneurons are moderately phase locked to LFP gamma for up to 3 spikes per theta stimulation period.

bioRxiv preprint doi: https://doi.org/10.64898/2026.04.21.719932; this version posted April 22, 2026. The copyright holder for this preprint (which was not certified by peer review) is the author/funder, who has granted bioRxiv a license to display the preprint in perpetuity. It is made available under aCC-BY 4.0 International license.

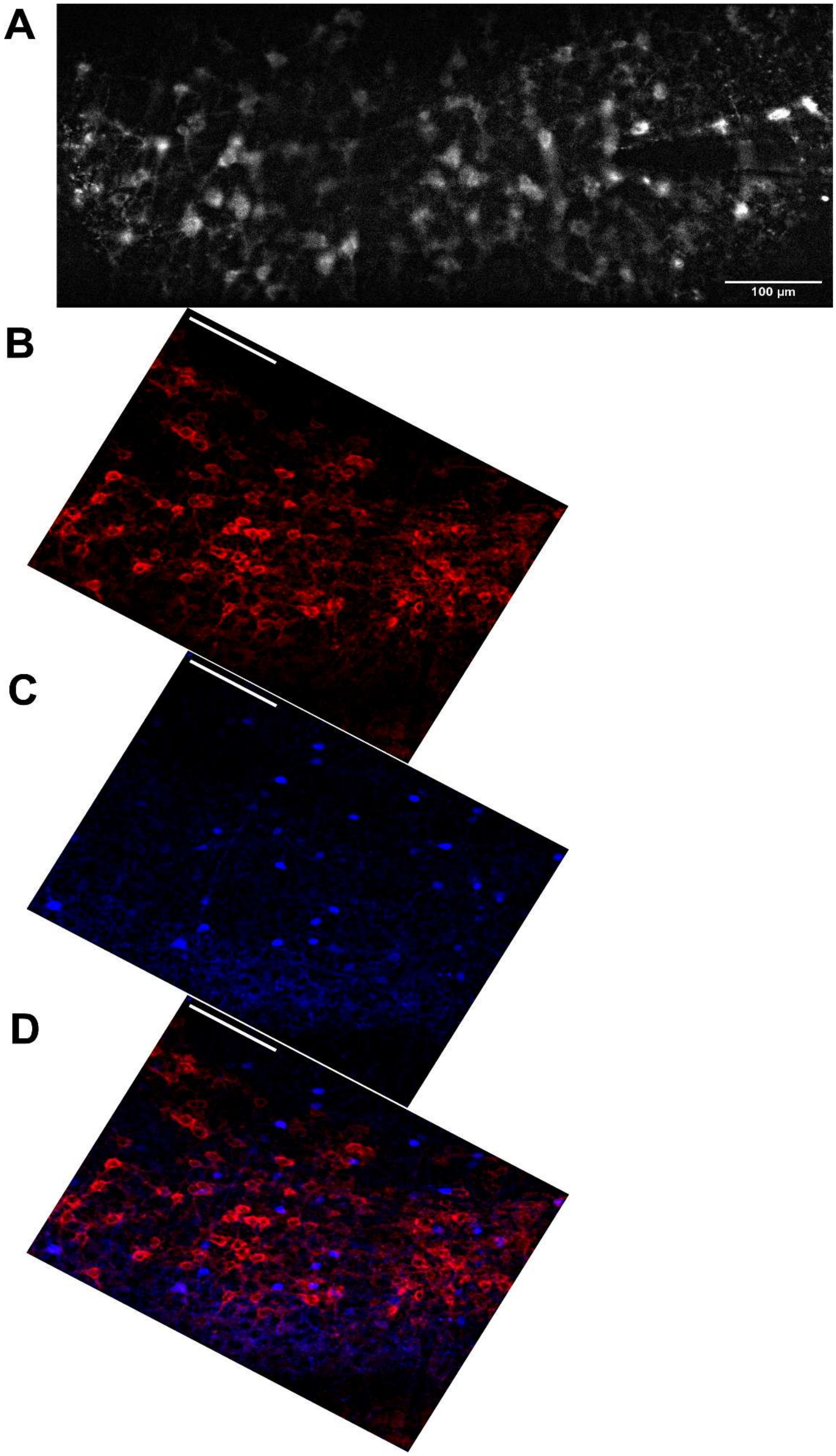

1103

bioRxiv preprint doi: https://doi.org/10.64898/2026.04.21.719932; this version posted April 22, 2026. The copyright holder for this preprint (which was not certified by peer review) is the author/funder, who has granted bioRxiv a license to display the preprint in perpetuity. It is made available under aCC-BY 4.0 International license.

Figure S2: Minimal PV+ overlap with Voltron2 expression in layer II/III mEC. A) Example voltage imaging field of view with dense Voltron2 expression. B) Histological image of Voltron2-JF585 fluorescence registered to experimental field of view. C) Immunohistological image of PV expression in the same field of view as A and B. D) Composite image of Voltron2-JF585 (red) and PV (blue) expression in layer II/III mEC demonstrating minimal overlap between the populations.

bioRxiv preprint doi: https://doi.org/10.64898/2026.04.21.719932; this version posted April 22, 2026. The copyright holder for this preprint (which was not certified by peer review) is the author/funder, who has granted bioRxiv a license to display the preprint in perpetuity. It is made available under aCC-BY 4.0 International license.

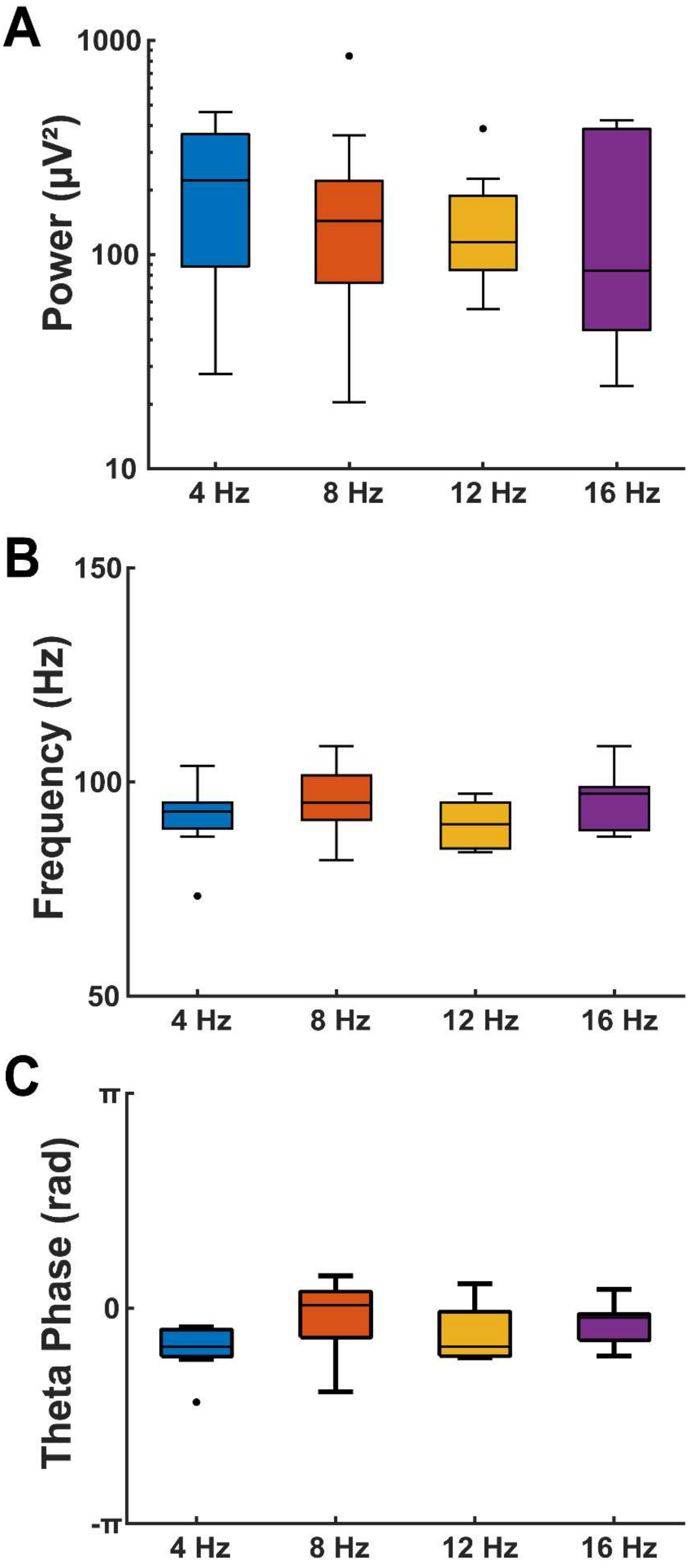

bioRxiv preprint doi: https://doi.org/10.64898/2026.04.21.719932; this version posted April 22, 2026. The copyright holder for this preprint (which was not certified by peer review) is the author/funder, who has granted bioRxiv a license to display the preprint in perpetuity. It is made available under aCC-BY 4.0 International license.

Figure S3: LFP gamma frequency activity doesn't change with theta stimulation frequency. A) Average scalogram peak LFP gamma power from each voltage imaging field of view. B) Average scalogram LFP gamma frequency. C) Average scalogram LFP theta phase of peak gamma power.

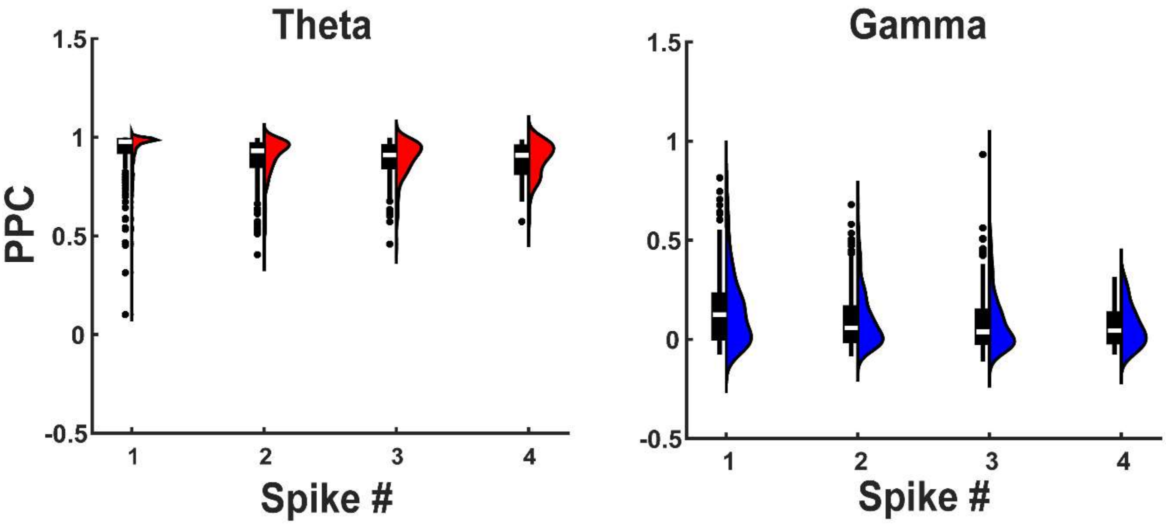

Figure S4: Strong theta (left), but not gamma (right) phase locking across multiple consecutive spikes per theta period from neurons in all imaging sessions.

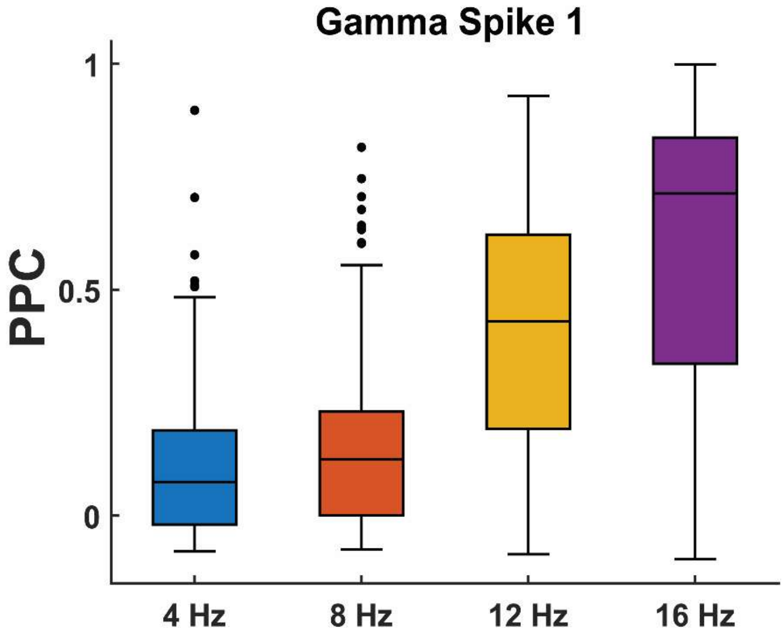

Figure S5: First spike gamma phase locking increases with theta stimulation frequency.

bioRxiv preprint doi: https://doi.org/10.64898/2026.04.21.719932; this version posted April 22, 2026. The copyright holder for this preprint (which was not certified by peer review) is the author/funder, who has granted bioRxiv a license to display the preprint in perpetuity. It is made available under aCC-BY 4.0 International license.

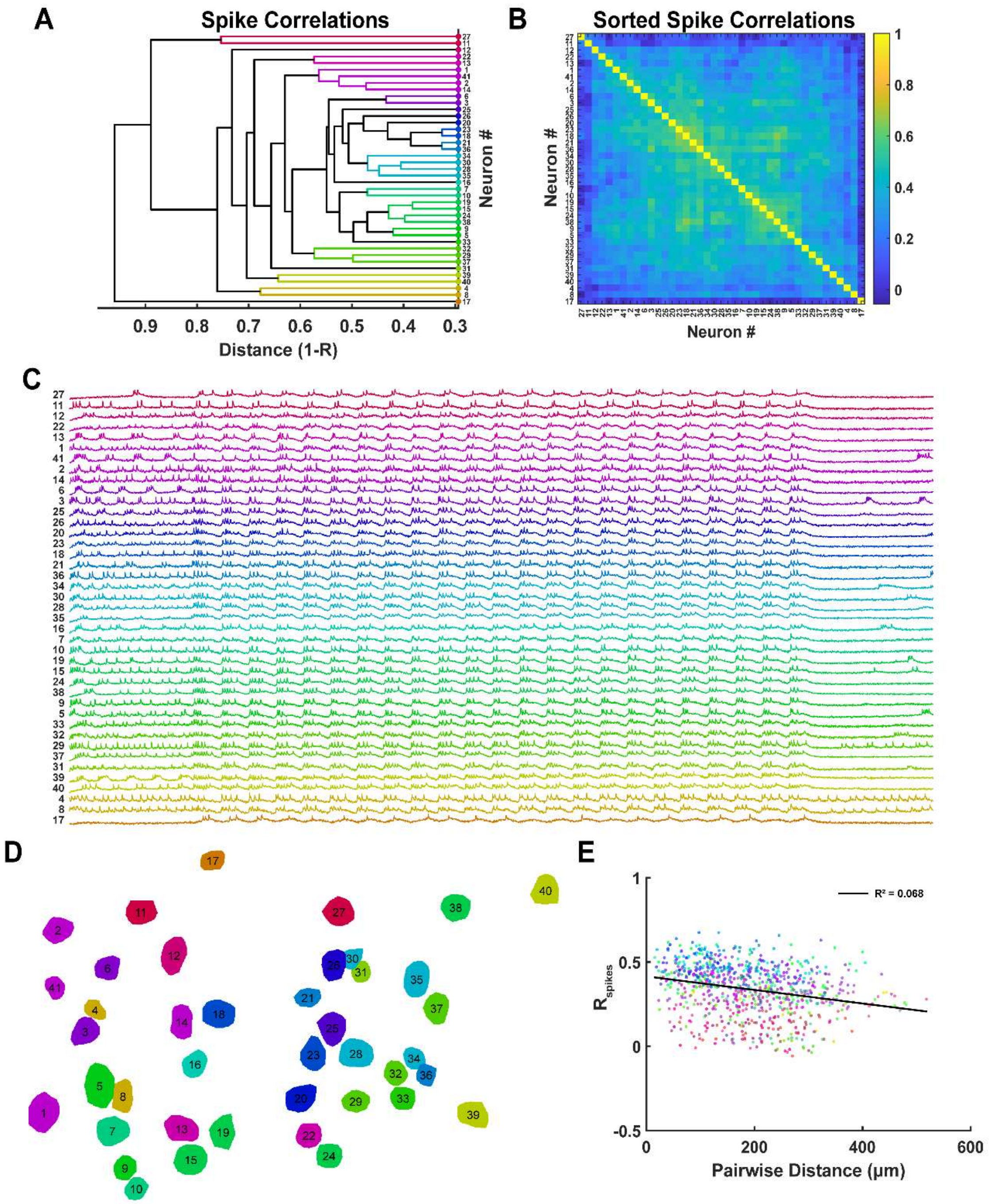

Figure S6: Spiking activity is not clustered across mEC. A) Agglomerative dendrogram of average spike correlation linkages between 41 mEC neurons in Fig. 5, 6. B) Sorted spike correlation matrix based on hierarchical clustering optimal leaf order. C) Sorted time series voltage activity based on hierarchical clustering of spike correlation matrix. D) Spatial organization of spike correlation clusters across the imaging field of view. E) Pairwise spike correlations are not correlated with distance.
